# Virtual Reality as an Adjunct Home Therapy in Chronic Pain Management: An Exploratory Study

**DOI:** 10.2196/medinform.7271

**Published:** 2017-05-11

**Authors:** Bernie Garrett, Tarnia Taverner, Paul McDade

**Affiliations:** ^1^ School of Nursing University of British Columbia Vancouver, BC Canada; ^2^ Faculty of Science University of British Columbia Vancouver, BC Canada

**Keywords:** pain management, chronic illness, therapeutics, medical informatics

## Abstract

**Background:**

Virtual reality (VR) therapy has been successfully used as an adjunct therapy for the management of acute pain in adults and children, and evidence of potential efficacy in other health applications is growing. However, minimal research exists on the value of VR as an intervention for chronic pain.

**Objective:**

This case series examined the value of VR to be used as an adjunctive therapy for chronic pain patients in their own homes.

**Methods:**

An exploratory approach using a case series and personal interviews was used. Ten chronic pain patients received VR therapy for 30 min on alternate days for 1 month. Pre- and postexposure (immediately afterwards, 3 h, and at 24 h) pain assessment was recorded using the Numerical Rating Scale (NRS), and weekly using the Brief Pain Inventory (BPI) and Self-completed Leeds Assessment of Neuropathic Symptoms and Signs pain scale (S-LANSS). Terminal semistructured personal interviews with the patients were also undertaken.

**Results:**

Of the 8 patients who completed the study, 5 of them reported that pain was reduced during the VR experience but no overall treatment difference in pain scores postexposure was observed. VR was not associated with any serious adverse events, although 60% of patients reported some cybersickness during some of the experiences.

**Conclusions:**

Of note is that the majority of these study participants reported a reduction in pain while using the VR but with highly individualized responses. One patient also reported some short-term improved mobility following VR use. Some evidence was found for the short-term efficacy of VR in chronic pain but no evidence for persistent benefits.

## Introduction

Research on virtual reality (VR) dates back to the early 1980s [1], but the potential for mainstream use has only recently been realized. Some successes have been reported in the use of VR in the treatment of acute pain as an adjunctive method for pain control [2-7]. Clinical studies exploring its use for chronic pain remain minimal [8-10]. Results have been hopeful, but some used artificially induced pain in healthy adults rather than actual chronic pain patients; thus, findings may not be clinically comparable [8]. Nevertheless, the value of VR in chronic pain management remains an area of potentially high impact research. In Canada, chronic pain is a significant health issue with 18.9% of adult Canadians suffering from chronic persistent pain [11]. Chronic pain persists as a complex phenomenon affecting millions of Canadians every day [11-15]. Chronic pain patients also often find their pain experiences persist despite medical interventions, and those affected frequently suffer from additional decreases in psychosocial health and activity restriction [16-17].

Cognitive factors are well-known to affect perceptions of pain [18]. Currently, VR environments are hypothesized to reduce pain via cognitive attentional and distractive mechanisms, although the exact mechanisms remain unclear [19-25]. The use of VR might act directly and indirectly on pain perception in a number of ways by altering signaling pathways involving attention, emotion, concentration, memory, touch, and the auditory and visual senses. VR interventions appear to reduce pain sensitivities by altering the sense of personal presence to that of being in new virtual environment, changing the sensory, affective, and cognitive features of the experience and altering the subjective perception of pain [26,27]. Therefore, the potential value of VR to help mediate chronic pain is an important area for exploration. The primary aims for this exploratory study were to identify any changes from baseline pain scores and in reported pain experiences using VR, and establish whether VR can be practically and safely used at home. Secondary aims included identifying any weekly pain score changes, any adverse effects [28], effects on function, and any preferences in type of VR experience. In addition, the study was undertaken to evaluate feasibility and establish practical methods to research VR interventions for chronic pain.

## Methods

### Design

A mixed-methods pilot case-series approach was used. The quantitative a priori hypotheses tested were that for patients with established chronic pain treated at home: (1) exposure to VR for 30 min 3 times a week would decrease pain scores from their preexposure baseline, and (2) exposure to VR sessions 3 times a week would lead to decreased weekly pain scores over a month. The qualitative aspects of the study examined patient’s perceptions of their pain while using VR, if they observed any practical application of safety issues in using VR or experienced any adverse effects. Also, their VR experience preferences, and if they noted any functional or quality-of-life improvements during the study were examined.

### Sample Selection and Recruitment

Prior to recruitment, a review of the proposal was undertaken by the UBC Clinical Research Ethics Board and approval granted. A purposeful nonprobability convenience sample of 10 adult patients with a diagnosed chronic pain condition for at least six months were recruited by Web-based invitations from 2 sources; PainBC and the People in Pain Network. Both are charitable support organizations, based in British Columbia. Interested patients were sent further information and a telephone interview by a member of the research team was conducted to answer the patients’ questions and screen them against specific inclusion and exclusion criteria (see Textboxes 1 and 2). Suitable patients were then sent an informed consent form to be signed and returned on installation of the VR equipment (with a duplicate copy provided for them to retain).

**Inclusion criteria for the study.**

• ≥18 years

• Have had a chronic pain diagnosis for 6 months or longer

• Score a maximum of ≥4 on the NRS pain scale daily

• Have desk space at home for the VR headset and accompanying computer system

• Able to understand the English language, and read and write English

• Able to wear a VR HMD (head-mounted display) and move head in cervical rotation, extension, and flexion

• sufficient fine motor control to operate a joystick/game controller

**Exclusion criteria for the study.**

• Individuals who have cognitive impairment or inability to control a basic computer VR interface, or complete questionnaires

• Susceptibility to motion sickness or cyber-sickness (LaViole 2001)

• Susceptibility to claustrophobia

• History of susceptibility to seizures

### Intervention

A home-based VR intervention was selected for the study. First, for practicality, as many of these patients also had mobility concerns and it was not economical to offer transportation to a hospital or lab and back again several times a week. Second, commercial VR systems would need to be suitable and easy to use at home, if they proved efficacious in the treatment of chronic pain. The VR hardware used was identical for each patient, and consisted of a high-end personal computer running the VR applications with an Oculus Rift DK2 110^0^ field of view (FOV) stereoscopic head-mounted display (HMD) with a resolution of 960 × 1080 pixels per eye.

**Figure 1:**
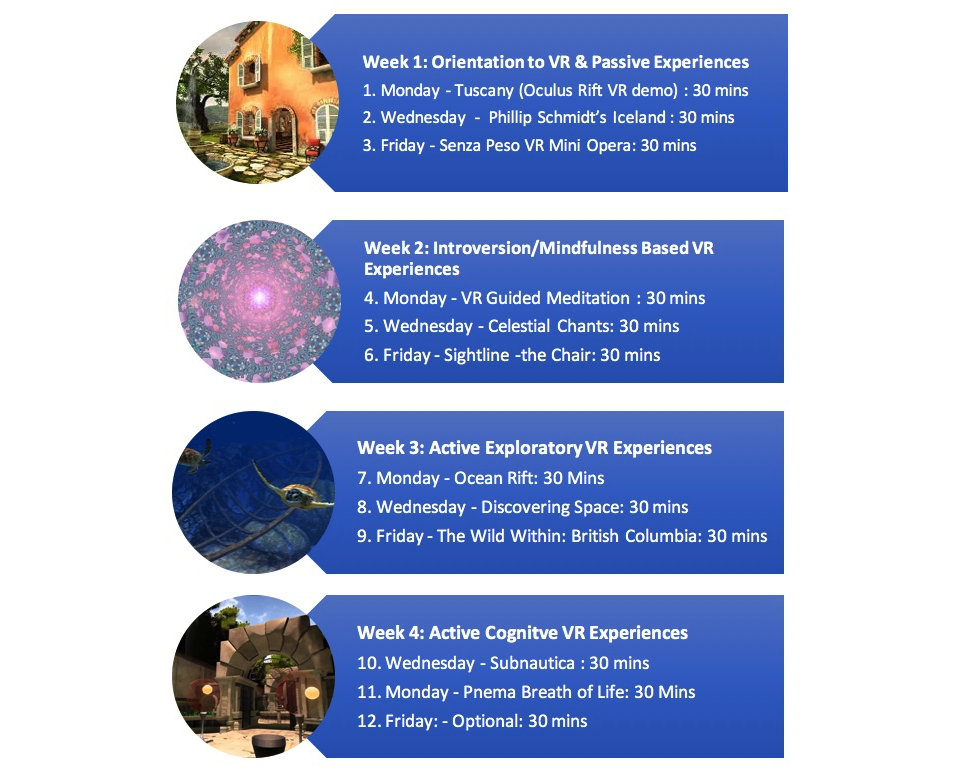
VR (virtual reality) intervention protocol and sequence.

As there were no VR experiences validated in the context of chronic pain, one of the project’s aims was to explore whether patients expressed preferences for different VR environments. Four categories of VR experience were devised, and VR applications were purposefully selected and tested in advance for potential efficacy by the researchers for use in each of the 4 weeks of the study. In week 1, the participants undertook passive VR experiences where they simply travelled through a VR environment. These included a virtual Iceland, and a boat ride through an artistic experience (Senza Peso). In the second week, mindfulness and meditative introversion focused VR applications were used, as these have been associated with pain control in other studies [29,30]. These experiences involved flying through 3D mandalas, or experiences that altered the user’s environment depending where they looked (Sightline). In the third week, active exploratory VR environments were used, where the participant could explore a new environment at will (an underwater environment, the solar system, and a natural environment). In the final week, active problem-solving experiences were used (eg, game type environments requiring participants to solve 3D puzzles). These different applications allowed for comparison of the VR environments in terms of any reported specific effects on patient’s pain experiences and side effects, and also prevented boredom with the VR experiences available at the time. An identical protocol of specific VR experiences to be used for 30 min on every other day of the study was given to each patient (see Figure 1). A simple computer menu system was devised so that patients could easily start each VR application on the appropriate day in sequence (as per protocol). Daily VR activity was logged, with arrest from VR at weekends.

Three sets of equipment were used concurrently with different patients, and then moved on to the next patients who had volunteered on a previously arranged schedule. The equipment was installed in the patients’ home with a 90-min training session on how to use the VR and perform data collection procedures. Patients were also assessed as being able to navigate the VR experiences and use the equipment comfortably at the end of this. During the study time patients also had access to a member of the research team by telephone and email for trouble shooting and to discuss any issues associated with the study.

Given an absence of prior work with VR and chronic pain, an initial exposure to 12 therapeutic sessions of 30 min was identified as reasonable to explore the clinical effects of VR initially. VR research in acute pain settings was usually of 15-30 min duration [2] and in associated pain hypnotherapy studies that used 4-6 sessions had less success whereas studies that had offered 8-12 sessions with lengths of treatment exposure between 30 and 40 min established positive results [31,32].

### Instruments: Quantitative Tools

To ensure the multidimensional aspects of chronic pain experienced by individuals were adequately measured, various tools were selected that addressed different aspects of chronic pain. These were self-recorded by patients in a supplied binder.

#### Pre- and Postexposure NRS Scores

Participants were asked to self-rate and record their pain intensity in a diary using the NRS immediately before and after the intervention and at 6 and 24 h postintervention (it was impractical to record pain during the VR experience as this would have proved disruptive to the experience). These measurement points supported the capture of any residual therapeutic effects or trends following the intervention. The NRS meets the IMMPACT group recommendations for using a global impression of change question [33,34].

#### Weekly Pain Trends: The Brief Pain Inventory (BPI) and Short Leeds Assessment of Neuropathic Symptoms and Signs (S-LANSS)

The BPI and the S-LANSS were used initially (as a baseline) and then at the end of each week of therapy to capture more detailed pain assessment data (giving scores recorded at 5 different time points). The BPI is a validated tool that has been used in numerous studies investigating pain [35-37]. The S-LANSS was specifically designed to measure the pain qualities associated with neuropathic pain and treatment effects. It is a quick to use self-reporting scale consisting of 12 distinct questions, which ask about the intensity and quality of the patients’ pain [38].

#### Cybersickness Reporting Form

Cybersickness is a well-documented side-effect of VR experiences [28,39-41]. It is the tendency for some users to display symptoms analogous to motion sickness both during and after the VR experience. It is distinct from motion sickness, in that the user is normally stationary but has a compelling sense of self-motion through moving visual imagery. A simple guide to avoid cybersickness and self-reporting form was provided for patients to record any episodes on a weekly basis, indicating the experiences that had led to the episode, onset and duration, any factors that alleviated the sickness, and also any departures from the protocol provided.

### Instruments: Qualitative Tools

#### Initial Individual Interviews

A short semistructured personal interview with each patient was executed at the start of the study by a researcher to capture basic biographic information from participants and a pain history (eg, ethnicity, age, gender, and prior VR and computer gaming experience; pain history: cause, duration, onset, nature, treatment history, and current pharmacological and other interventions used for pain).

#### Terminal Individual Interviews

An audiotaped 30-min semistructured personal interview was undertaken at the end of the study by a researcher during the final visit to collect the VR equipment. The interview was designed to capture the patient’s perceptions of the value of the VR interventions in the management of their pain, their overall impressions of the experience, and any adverse effects.

### Data Analysis

Quantitative data were explored for any differences in the average pain scores for the VR intervention at each time point to analyze for any indications of changes in pain immediately following the VR experience, and at 6 and 24 h after exposure, and for cybersickness. Descriptive univariate statistics were analyzed to support a preliminary understanding of the impact of VR on an individual’s pain experience and nature of the data obtained. A simple initial pre-post exposure NRS analysis was then undertaken using a Wilcoxon matched pairs test. For the BPI and S-LANSS scores, a Freidman test (the nonparametric alternative to the one-way repeated measure ANOVA) was undertaken to explore for any trends evident over the whole month using SPSS 23 statistical software (IBM).

Qualitative data were transcribed from the original sources into Nvivo 7.0 qualitative analysis software. It was analyzed using an interpretive-description (ID) approach for an open exploration of participant’s experiences to further understand the perceptions associated with the use of VR and any impact on their chronic pain. ID assumes preexisting theoretical knowledge, and that clinical patterns exist, and rather than trying to avoid preconceptions in the analysis, coding proceeds on the basis that no matter how participatory and collaborative the analytical method is, it will finally be the researcher who determines what data are significant [42,43]. An ID approach allowed for an inductive descriptive analysis of the phenomena, using iterative readings of the combined qualitative data and coding by 2 independent members of the research team. Analyses were then merged to establish key thematic elements, patterns, and theory associated with the patient’s experiences [42].

## Results

### Sample Characteristics

As is common with chronic pain studies, the study encountered attrition and 8 patients (n=8, 33% attrition) completed the full study protocol, and only 6 of these consented to post experience interviews [44,45]. Reasons for discontinuation were not required, although one indicated it was due to cybersickness. The patients’ pain conditions and histories are summarized in Table 1.

**Table 1:** Participant characteristics. All participants were unemployed, had high-school graduate education levels, and good computer literacy.

ID	Age	Gender	Pain diagnosis	Pain treatment history
01	48	Female	Low back and knee pain following traumatic injury 6 years ago.	Pharmacological: Acetaminophen & Codeine (Tylenol 3), Oxycodone & Acetaminophen (Percocet), Gabapentin, Ibuprofen, Diclofenac, Oxycodone.
			Reported daily NRS score: 4-6	Surgical: Right knee replacement.
				Other: Physiotherapy, Occupational therapy, Intramuscular Stimulation (IMS)
				
02	63	Female	Arachnoiditis and low back pain following traumatic injury 4 years ago.	Pharmacological: Acetaminophen & Codeine (Tylenol 3), Ibuprophen, Pregabalin, Gabapentin, Cortisone injection, Ketamine, Fentanyl patches, Hydromorphone.
			Reported daily NRS score: 4-7	Surgical: Microdiscectomy, Nerve block.
				Other: Physiotherapy, Chiropractic
				
04	66	Male	Ilioinguinal neuralgia following hernia repair 14 years ago. Ankylosing Spondylitis over the last 6 years.	Pharmacological: Acetaminophen & Codeine (Tylenol 3), Ibuprophen, Pregabalin, Gabapentin, Ketamine, Methadone, Lignocaine (topical), Oxycodone.
			Reported daily NRS score: 4-8	Surgical: Inguinal surgical mesh removal and inguinal neurectomy.
				Other: Physiotherapy, Yoga
				
05	50	Male	Cervical spine and shoulder pain following traumatic injury 20 years ago.	Pharmacological: Gabapentin, Clonazepam, Nabilone, Oxycodone, Magnesium Injection, Buprenorphine patches.
			Reported daily NRS score: 5-8	Other: Physiotherapy, Occupational Therapy, Massage, Acupuncture, Water Therapy, Myofascial Release (MFR)
				
08	71	Female	Chronic hip & lower back pain for 20 years from strain caused through professionally playing classical guitar for 30 years.	Pharmacological: Acetaminophen & Codeine (Tylenol 3), Ibuprophen, Pregabalin.
			Reported daily NRS score: 4-8	Other: Meditation, Naturopathy (prescribed Turmeric)
				
09	31	Female	Complex regional pain syndrome (Type 2) secondary to thrombosis and multiple embolism 6 years ago	Pharmacological: Acetaminophen & Codeine (Tylenol 3), Naproxen, Cyclobenzaprine, Gabapentin, Hydromorphone, Pregabalin, Fentanyl patches.
			Reported daily NRS score: 6-9	Other: Physiotherapy, Massage Therapy, Intramuscular Stimulation (IMS)
				
10	43	Female	Migraine headaches and small fiber myopathy following traumatic injury and resulting brain lesion when 7 years old.	Pharmacological: Topiramate, Pregabalin, Cannabis vaporizer and Buprenorphine patch.
			Reported daily NRS score: 5-7	Other: Acupuncture, Chiropractic, Massage therapy
				
11	36	Female	Low back pain and myofascial pain following traumatic injury 8 years ago	Pharmacological: Acetaminophen & Codeine (Tylenol 3), Ibuprofen, Baclofen, Lidocaine/Ketamine cream, Gabapentin, Pregabalin, Lidocaine injection, Tramadol Reported daily NRS score: 5-8 & Acetaminophen (Tramacet).
				Other: Physiotherapy, Occupational Therapy, Yoga, TENS

### Quantitative Analysis

**Figure 2:**
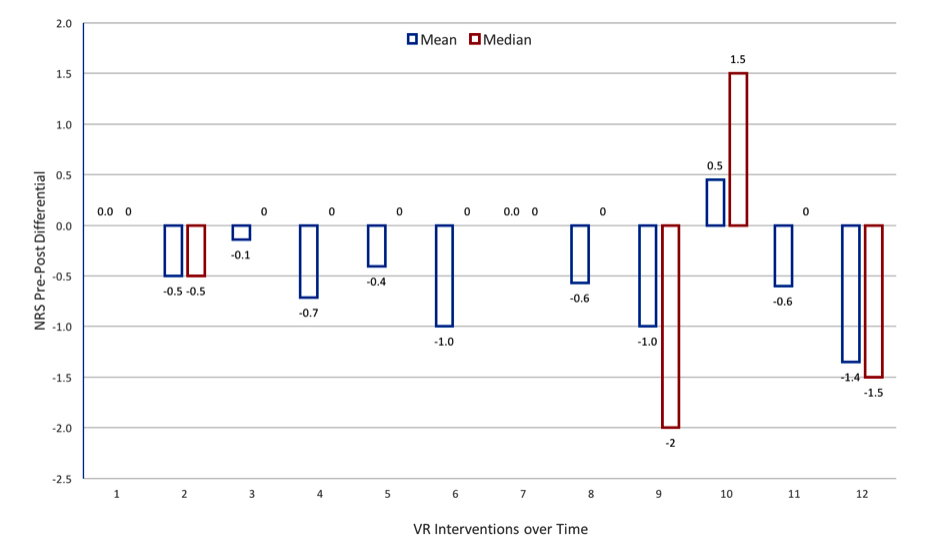
Pre-postexposure NRS score differentials by intervention. NRS: Numerical Rating Scale.

A descriptive statistical exploration of the various pain scores confirmed the data were not normally distributed. Univariate comparison of the mean NRS pre-post test scores demonstrated a slight decrease in pain for most interventions (see Figure 2), although for one intervention (#10) a slight increase in pain was also observed. This likely reflected user frustration with the Subnautica prerelease app, which had not yet implemented game-controller functionality, so participants had to move around using keyboard controls, which was difficult with an HMD on. No reduction in NRS scores 6 and 24 h later was evident. Furthermore, a Wilcoxon Matched Pairs Signed Ranks test for means for each VR intervention demonstrated no significant effect between pre- and postexposure for NRS scores (see Table 2). Given the lack of any significant pre-post exposure effects, no further exploration of NRS scores was performed. The Friedman test run on the BPI and SLANNS scores as repeated measures for each participant at the end of each week also indicated no statistically significant difference in the pain reported over the 4 weeks. BPI Worst Pain: x^2^=1.6, *P*=.82 BPI Average Pain: x^2^=5.2, *P*=.27; BPI Least Pain: x^2^=4.6, *P*=.20. BPI Pain Now: x^2^=2.9, *P=*.57. S-LANSS x^2^= 1.0, *P*=.91. As no significant findings were obtained, no further post hoc analysis was performed.

**Table 2:** NRS scores pre-post VR exposure Wilcoxon signed ranks test.

Value	Post1 - Pre1	Post2 - Pre2	Post3 - Pre3	Post4 - Pre4	Post5 - Pre5	Post6 - Pre6	Post7 - Pre7	Post8 - Pre8	Post9 - Pre9	Post10 - Pre10	Post11 - Pre11	Post12 - Pre12
Z	.000^a^	−1.414^b^	−.322^b^	−1.633b	.000^a^	−1.841^b^	.000^a^	−.680^b^	−1.289^b^	.000^a^	−.816^b^	−.816^b^
Asymptotic Significance (2-tailed)	>.99	.16	.75	.10	>.99	.07	>.99	.50	.20	>.99	.41	.41

^a^The sum of negative ranks equals the sum of positive ranks.

^b^Based on positive ranks.

### Qualitative Analysis

Four major thematic areas emerged from the interview analysis: design of the VR experiences, efficacy of VR for chronic pain, limits of the VR technology, and practicality of use as an adjunctive therapy (see Table 3). The subthemes evident in these are described below together with participant quotes.

There was a distinct difference in participants’ perceptions of the value of VR environments designed to be interactive versus those designed to promote relaxation. Half of the interviewees believed that the interactive experiences were more beneficial. For example:

The space one too, when you’re exploring and you’re driving, I loved that one too. I thought that was so much fun, I love the ones when you using your brain, and you’re actually trying to do stuff, I enjoyed those more, but when I was done, I didn’t even notice.

Participant #10

…because then you are actually immersed in it, whereas some of the ones you felt like even though it was 3D or whatever, you weren’t really immersed yeah you were a recipient of the experience, but the ones that made you think and do and that and react were more immersive and then the more immersive it is the more it worked.

Participant #1

Two participants reported they actively disliked the relaxation-based VR experiences:

I didn’t feel that um, I didn’t sense that just floating over Iceland or um, some of those other things didn’t do it for me

Participant #4

**Table 3:** Key themes emerging from Interviews (numbers=number of separate responses).

Major theme	Subtheme (positive)	Subtheme (negative)
VR design	Relaxation (7)	Relaxation anxiety (3)
	Interaction (17) Immersion (3)	
	Variety (6)	
	Enjoyment (4)	
Efficacy	Effective (14)	Ineffective (4)
	Distraction (8)	Transient (10)
	Mobility (2)	Stressful (3)
Technological functionality	Comfort (1)	Frustration (13)
		Technical issues (12)
		Graphics quality (4)
		Comfort (2)
Practicality	-	Side effects: Cybersickness (6) Claustrophobia (1)
		Engagement (5)
		Position (3)

However, 3 participants favored the relaxing experiences as in:

…some programs were really good like the guided meditation one I found really good, managed to make me feel really relaxed by the end of it.

Participant #9

There appeared no relationship between the type of VR experience people preferred and types of pain they experienced.

The second theme related to the efficacy of the VR experiences in helping reduce pain. Four of those interviewed identified positive benefits during the VR experience:

when I was doing them I didn’t notice the pain until I was done. So, when I play the games, no pain. But when I filled-in the questionnaire, that’s when I noticed it!

participants #1, 4, 8, and 10

Participants who experienced positive effects, distraction was frequently highlighted as the rationale:

So overall as an experience, if the idea is a distraction to the pain, yeah it worked for a little while then.

Participant #4

…actually take your mind off the world, cause you’re in a totally different place, a fantasy.

Participant #10

All the participants who found benefits, all reported the experience was transient and did not persist long after the VR experience:

The effect on my pain wore off and I was aware of my pain certainly within a few hours of using the VR.

Participant #1

Two participants reported the VR experiences were not effective for helping reduce their pain:

Overall I would just say it was really interesting, versus like actually helping with my pain.

Participant #11

One participant (#10) who had reported positive effects with some of the VR experiences noted one actually caused her stress, increasing her pain:

Where I was under water in the ocean, I saw the shark, and I thought it was going to attack me. It was giving me anxiety a bit, and stressing me out a bit, so as soon as I saw that my hand started burning, and my feet…

Participant 10

Although conversely, another acknowledged:

Some were more stressful…but I find that - when I get stress my pain doesn’t increase…

Participant #11

The technological functionality (and limits) of the available VR experiences also emerged as noteworthy. Four participants identified that they became quite frustrated with the VR systems in use, because of complex or cumbersome control systems:

I had some trouble figuring out which controls to use to move around so um I’ve never played computer games before and maybe that had something to do with it. I felt like a total idiot totally frustrated and not able to catch onto what to do.

Participant #8

The comfort of the HMD drew both positive and negative comments. One user believed that the HMD was *“…nice and comfortable it was relatively small.”* (Participant #1), whereas 2 others complained about issues trying to use the HMD with eyeglasses:

It was a bit uncomfortable working with my glasses.

Participant #4

Similarly, another theme on the practicality of using VR was evident. The side effect of cybersickness was significant and reported by 5 of the 8 participants (60%) for a least one VR experience in the self-reporting forms, and it also arose as a topic of interest in the interviews:

There was a few times where I had to stop because I felt sick because of how fast I felt I was going.

Participant #11

Most of the responses noted mild nausea being induced when experiences involved rapid speed or motion, or moving in 3 dimensions (such as in the spacecraft simulator), which was resolved when they slowed the experience down or had a break. However, 1 participant noted the symptoms persisted for some time after the VR experience:

I slowed down so I felt a little less bad and I thought I could continue. But afterwards I went and my mom got me a ginger ale, and I laid down and I thought, thank goodness I don’t have that in the car or whatever.

Participant #11

Two participants also noted minor claustrophobia as a side effect:

I felt a bit of claustrophobia because when I was under water and I realized at first I didn’t know how to get above the water and was running out of air.

Participant #4

The issue of being able to engage with a VR experience when the participant was experiencing severe pain also arose as a practical limitation. In 2 cases, they reported they were in too much pain to use the VR equipment.

## Discussion

### Principal Findings

Although there were no significant pre-post exposure changes in the reported pain scores, more than half (5 of 8) of the participants did report positive benefits on their perceived pain from the use of VR. However, 3 of the participants interviewed reported none. The effects of VR on chronic pain would appear to be very individualized. No evidence that any benefits of using the VR on the participant’s pain persisted postexposure was found. For participants who identified positive results, distraction was described as the mode of action by them. This is consistent with other researchers who suggest the deeper form of distraction produced with VR experiences is the main mechanism by which pain is attenuated [19,20,22,31,46].

Chronic pain patients respond in very individualistic ways to VR as indicated by the varying preferences for interactive versus relaxing forms of VR reported. Some had very negative reactions to the relaxation introversion-focused experiences, whereas others enjoyed them. This finding is consistent with work that reports some individuals are actually relaxation-sensitive and paradoxically find relaxing experiences increase their stress levels [47,48]. Although no significant improvement in BPI and functionality was evident over time, one of the participants did report improved mobility following the VR experiences. Functional improvement has also been reported in other VR studies [49-51].

It was evident that VR technology remains immature in the technology life cycle, the progression from research and development, commercial production, to succession by superior technologies [52,53]. Most participants reported some technical issues with either the hardware or software during the experience. However, the possibility of achieving nonpharmacological pain relief may encourage more chronic pain suffering to experiment with VR, as they are more likely to experiment when pharmacological and other medical therapies fail [54,55].

The most significant adverse reaction to VR was cybersickness, as 60% of the participants experienced this at some time during the study. This side effect of VR is well documented, but should not be underestimated as a factor that may influence uptake [28,39-41,56-59]. Other than cybersickness, no significant adverse effects were noted.

### Limitations

As an initial exploratory study, this work has limitations. Case series are vulnerable to selection bias and may not represent the wider population, and with small-scale studies such as this, the effects seen may be due to intervening effects such as the placebo, Hawthorne, or Rosenthal effect. Therefore, internal validity and reliability may be limited. Technological immaturity of the experimental setting is also a limitation. Strengths of the study include the inductive ID approach, which is more responsive to clinical experience-based questions here. Furthermore, an exploratory pilot study provides an appropriate approach at this fundamental stage of clinical research to inform future work.

Work in this area is in its infancy and clinical studies limited. The complexities of chronic pain make finding pain-management solutions challenging, and the results reflect those complexities. The following conclusions were drawn from the results:

Exposure to VR for 30 min a day every other day for chronic pain patients in self-administered therapy sessions resulted in:

- 66% of participants reporting a reduction in pain while using the VR therapy,
- No significant pre- or postexposure differential in pain scores,
- No significant postexposure impact on pain levels,
- No significant postexposure impact on pain interfering with daily function,
- 60% of patients reported episodes of cybersickness when using VR.

The majority of this study participants reported a reduction in pain while they were using the VR, but with highly individualized responses. Findings, as with other recent work suggests that VR maybe a useful short-term adjunct for the management of chronic pain, but individual choice in the form of VR experience may be as significant as the VR medium itself [60]. Although statistically significant reductions in pain scores postexposure were not demonstrated, in the qualitative analysis, participants reported mostly positive impacts on their pain experience, and one reported that the VR experience did appear to improve their mobility. Whereas longer-term benefits of VR therapy as an adjunctive for chronic pain were not demonstrated, the immediate relief experienced by patients here during VR would indicate the therapy has potential for a means of providing respite from the constant pain they experience.

### Conclusions

Attention to practical implementation is important, particularly having good orientation practices and technical support available to patients at home. Robust controlled trials with larger samples, comparing VR with other forms of multimedia and neurological studies are now required to establish efficacy. Practical methods to research VR interventions should include both active and passive interventions, and larger cohort studies including assessment of cybersickness (and factors that ameliorate its effects) as the most significant adverse effect.

In conclusion, home-based VR therapy is a feasible option for chronic pain sufferers. There remains a pressing need for non-opioid alternatives in the treatment of chronic pain, and in light of the patient’s experiences documented here, individual tailored VR solutions would appear more likely to be successful compared with a unidimensional off-the-shelf VR experience.

## Abbreviations

FOV: field of view
HMD: head-mounted display
VR: virtual reality
NRS: Numerical Rating Scale
S-LANSS: Self-completed Leeds Assessment of Neuropathic Symptoms and Signs pain scale
